# Imperatorin as a Promising Chemotherapeutic Agent against Human Larynx Cancer and Rhabdomyosarcoma Cells

**DOI:** 10.3390/molecules25092046

**Published:** 2020-04-28

**Authors:** Aneta Grabarska, Krystyna Skalicka-Woźniak, Michał Kiełbus, Magdalena Dmoszyńska-Graniczka, Paulina Miziak, Justyna Szumiło, Ewa Nowosadzka, Krystyna Kowalczuk, Sherief Khalifa, Jolanta Smok-Kalwat, Janusz Klatka, Krzysztof Kupisz, Krzysztof Polberg, Adolfo Rivero-Müller, Andrzej Stepulak

**Affiliations:** 1 Department of Biochemistry and Molecular Biology, Medical University of Lublin, 20-093 Lublin, Poland; michal.kielbus@umlub.pl (M.K.); magdalena.dmoszynska-graniczka@umlub.pl (M.D.-G.); paulinamiziak@umlub.pl (P.M.); ewa.nowosadzka@umlub.pl (E.N.); krystyna.kowalczuk@umlub.pl (K.K.); adolfo.rivero-muller@umlub.pl (A.R.-M.); andrzej.stepulak@umlub.pl (A.S.); 2 Independent Laboratory of Natural Products Chemistry, Medical University of Lublin, 20-093 Lublin, Poland; krystynaskalickawozniak@umlub.pl; 3 Department of Clinical Pathomorphology, Medical University of Lublin, 20-090 Lublin, Poland; j.szumilo@umlub.pl; 4 College of Pharmacy, Gulf Medical University, P.O. Box 4184 Ajman, UAE; sherief@gmu.ac.ae; 5 Department of Clinical Oncology, Holy Cross Cancer Center, 25-734 Kielce, Poland; jolantasmok1@gmail.com; 6 Department of Otolaryngology and Laryngological Oncology, Medical University of Lublin, 20-954 Lublin, Poland; januszklatka@umlub.pl (J.K.); krzysztof.kupisz@umlub.pl (K.K.); 7 Department of Otolaryngology, Center of Oncology of the Lublin Region St. Jana z Dukli, 20-090 Lublin, Poland; 8 Department of Otolaryngology, MSWiA Hospital, 20-331 Lublin, Poland; kpolberg@poczta.onet.pl; 9 Faculty of Science and Engineering, Cell Biology, ÅboAkademi University, 20520 Turku, Finland

**Keywords:** *Angelica archangelica*, *Pastinaca sativa*, furanocoumarins, imperatorin, rhabdomyosarcoma, larynx cancer

## Abstract

Naturally occurring coumarins are bioactive compounds widely used in Asian traditional medicine. They have been shown to inhibit proliferation, induce apoptosis, and/or enhance the cytotoxicity of currently used drugs against a variety of cancer cell types. The aim of our study was to examine the antiproliferative activity of different linear furanocoumarins on human rhabdomyosarcoma, lung, and larynx cancer cell lines, and dissolve their cellular mechanism of action. The coumarins were isolated from fruits of *Angelica archangelica* L. or *Pastinaca sativa* L., and separated using high-performance counter-current chromatography (HPCCC). The identity and purity of isolated compounds were confirmed by HPLC-DAD and NMR analyses. Cell viability and toxicity assessments were performed by means of methylthiazolyldiphenyl-tetrazolium bromide (MTT) and lactate dehydrogenase (LDH) assays, respectively. Induction of apoptosis and cell cycle progression were measured using flow cytometry analysis. qPCR method was applied to detect changes in gene expression. Linear furanocoumarins in a dose-dependent manner inhibited proliferation of cancer cells with diverse activity regarding compounds and cancer cell type specificity. Imperatorin (IMP) exhibited the most potent growth inhibitory effects against human rhabdomyosarcoma and larynx cancer cell lines owing to inhibition of the cell cycle progression connected with specific changes in gene expression, including *CDKN1A*. As there are no specific chemotherapy treatments dedicated to laryngeal squamous cell carcinoma and rhabdomyosarcoma, and IMP seems to be non-toxic for normal cells, our results could open a new direction in the search for effective anti-cancer agents.

## 1. Introduction

For many years, plants have been important sources of bioactive compounds widely used in medicine [1]. One of the largest group of phytochemicals, which possess many potential therapeutic properties, are naturally occurring coumarins. These substances are mainly classified based on their chemical structure into simple coumarins, linear and angular furanocoumarins, and pyranocoumarins [2].

Furanocoumarins are found in the *Apiaceae*, *Rutaceae*, *Moraceae*, and *Leguminosae* families [3] and show a wide range of biological activities [4]. Linear furanocoumarins called psoralens are well known as photosensitizing agents, which have been used in PUVA (psoralens plus UV-A) therapy for the treatment of autoimmune or hyper-proliferative skin diseases such as psoriasis and vitiligo [5]. Moreover, furanocoumarin/ultraviolet therapy known as photopheresis has become an effective treatment of cutaneous T-cell lymphoma [6]. Linear furanocoumarins have also been described to have antimicrobial, antioxidant, anti-inflammatory, antidiabetic [7], and anticolvulsant [8,9] activities.

Linear furanocoumarins have drawn attention in recent years as potential anti-cancer agents, either alone or in combination with other drugs. It has been shown that xanthotoxin and bergapten, independently of photoactivation, inhibit the growth of neuroblastoma, colon cancer cells [10], melanoma [11], hepatoma [12], and breast cancer cells [13]. Furanocoumarins such as imperatorin and bergamottin have been found to significantly enhance the cytotoxicity of cisplatin to hepatocellular carcinoma (HCC) cells [14] and potentiate the apoptotic effects of bortezomid and thalidomide in multiple myeloma (MM) cells [15]. It was also observed that psoralen sensitized lung (A549) and breast (MCF-7) cancer cells to docetaxel and adriamycin treatment, respectively. Psoralen suppressed P-glycoprotein function [16] and its expression at the mRNA and protein levels [17], thus reversing the multidrug resistance phenotype of lung cancer cells. Many coumarins serve as the chemical backbone for semi-synthetic derivatives under considerations as new anti-cancer drugs.

High-performance counter-current chromatography (HPCCC) was used as an efficient tool for isolation of both imperatorin (IMP) and xanthotoxin (XN) from plant material. The technique utilizes two immiscible phases, one as the stationary phase (retained in a spinning coil by centrifugal forces) and the second as the mobile phase and, owing to the lack of a solid stationary phase, it benefits from a number of advantages when compared with the more traditional liquid–solid separation methods. No irreversible adsorption, low risk of sample denaturation, total recovery, low solvent consumption, and ability of crude extract injection are only few of them. The HPCCC technique permits very high injection loadings, can be rapidly scaled from analytical to pilot level, and enables higher flow rates so that separation times are measured in minutes rather than hours at the same resolution [18]. As a source of target compounds, two widely occurring plants—*Angelica archangelica* L. and *Pastinaca sativa* L. (Apiaceae)—were selected. *A. angelica*, with imperatorin as a main compound, is believed to have ‘angelic’ healing power. This plant has been used in traditional medicine forages as a cure for central nervous system disorders, fever, skin problems, rheumatism, and different digestive problems. Much attention has so far been paid to the study of the antitumour and anticancer properties of various Angelica species, and most of these activities are primarily associated with the presence of various coumarins. Nowadays, powdered parts of the plant, essential oil, or extracts can be found in commercially available products on the market [19]. *P. sativa* is a vegetable with high nutritional value and dietetic quality. The plant has been used widely in European traditional medicine, mainly for its impact of digestive track, but also as a remedy for different central nervous system disorders [9]. As main coumarins, xanthotoxin and bergapten are indicated.

In the present study, we examined the antiproliferative activity of different linear furanocoumarins including imperatorin (IMP), isopimpinellin (IPP), xanthotoxin (XN), and xanthotoxol (XNO). Among these compounds, IMP exhibited the most potent growth inhibitory effects against human rhabdomyosarcoma and larynx cancer cell lines. Therefore, we further focused on its cellular and molecular mechanism of action.

## 2. Results

### 2.1. IMP Exhibits no Cytotoxic Effects to Normal Human Skin Fibroblasts (HSF) and Significantly Reduces the Viability of Human Rhabdomyosarcoma (TE671) and Larynx Cancer (RK33) Cells

**Figure 1:**
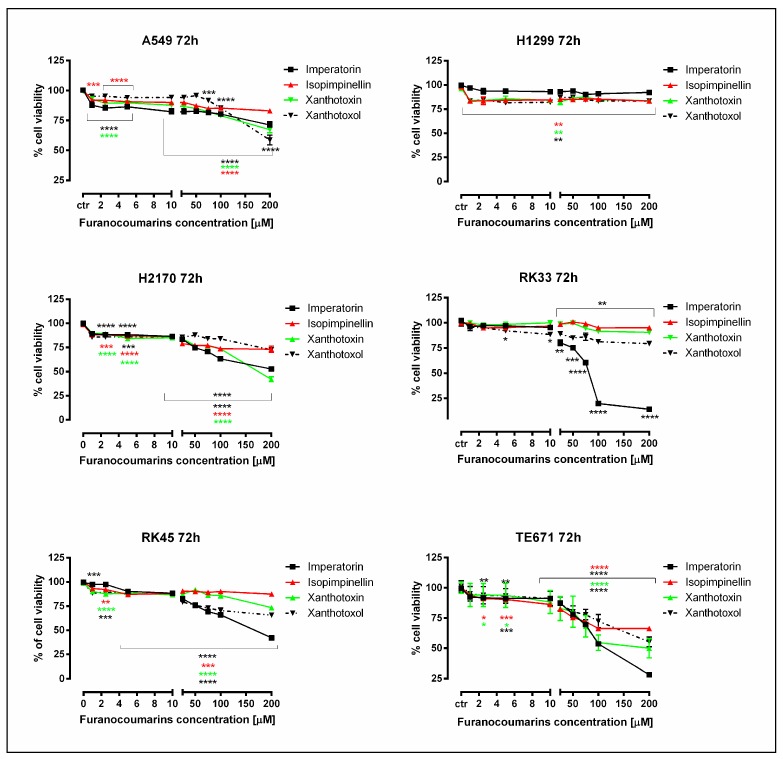
Antiproliferative effect of furanocoumarins: imperatorin, isopimpinellin, xanthotoxin, and xanthotoxol in human lung cancer (A549, H2170 and H1299), larynx cancer (RK33 and RK45), and rhabdomyosarcoma (TE671) cells. The cancer cells were exposed to either culture medium alone (control) or furanocoumarins (1–200 µM) for 72 h. Normalized cell viability measured by the methylthiazolyldiphenyl-tetrazolium bromide (MTT) assay is presented as mean ± SEM at each concentration. Tukey’s test revealed significant effect (* *p* < 0.05; ** *p* < 0.01; *** *p* < 0.001; **** *p* < 0.0001) of treatment with furanocoumarins compared with the control group. This effect was also concentration-dependent, as judged by analysis of variance (ANOVA) test (**** *p* < 0.0001), *n* = 24 per concentration from three independent experiments.

The antiproliferative activity of linear furanocoumarins on studied cancer cell lines was examined by methylthiazolyldiphenyl-tetrazolium bromide (MTT) assay. The cell lines selected for this study, including human lung cancer cell lines (A549, H2170, and H1299), larynx cancer cell lines (RK33 and RK45), and rhabdomyosarcoma cell line (TE671), were exposed to either culture medium (control) or different concentrations of IMP, IPP, XN, and XNO (1.0 to 200 µM) for 72 h. As shown in Figure 1, various cancer cell types displayed differential responses when treated with furanocoumarins.

Among these compounds, IMP significantly decreased cell viability in human rhabdomyosarcoma and larynx cancer cell (RK33) lines with IC_50_ (50% inhibitory concentration) values of 111.2 µM and 67.8 µM, respectively. RK33 cells were more sensitive to IMP than RK45 cells. The percentage of viable RK33 and RK45 cells after treatment with the highest concentration of IMP (200 µM) was 14.13% (**** *p* < 0.0001) and 42.18% (**** *p* < 0.0001), respectively. Moreover, the inhibitory effect of 200 µM IMP on the growth of studied lung cancer cell lines was more relevant on H2170 cells (52.73% of viable cells; **** *p* < 0.0001) than on A549 and H1299 cell lines (71.24% of viable cells; **** *p* < 0.0001 and 92.30% of viable cells; *p* > 0.05, respectively). Additionally, IPP, XN, and XNO also showed a weak effect on survival of H1299 cell line, which is derived from lymph node metastasis. XN was also found to exhibit mild antiproliferative activity against H2170 (IC_50_ = 196.8 µM) and TE671 (IC_50_ = 200 µM) cells, respectively. The final concentration of dimethyl sulfoxide (DMSO, the solvent) in the culture medium did not exceed 0.1% and did not influence the assays (data not shown).

**Figure 2:**
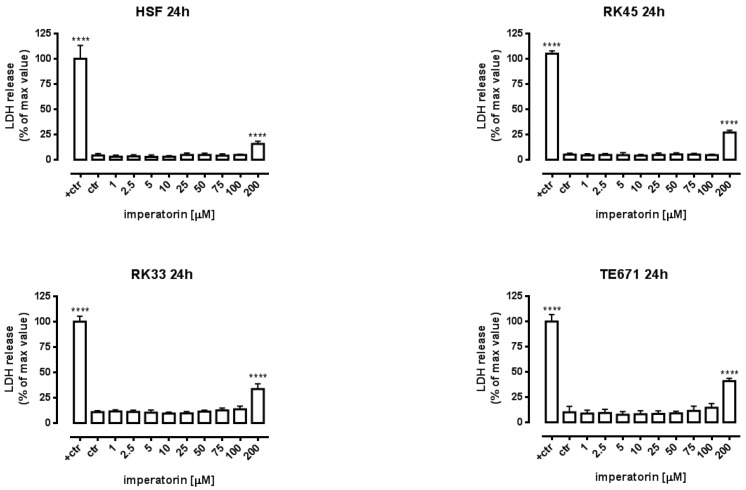
Cytotoxicity of imperatorin to normal human skin fibroblasts (HSFs), larynx cancer (RK33 and RK45), and rhabdomyosarcoma (TE671) cells. Lactate dehydrogenase (LDH) ELISA kit was used to quantify cytotoxicity by measuring LDH activity released from damaged cells. HSFs were incubated for 24 h alone or in the presence of imperatorin (1–200 µM). The results are presented as the percentage in LDH release to the medium by treated cells versus cells grown in control medium (ctr) and cells treated with lysis buffer (ctr+). Data are presented as mean mean ± SEM at each concentration. **** *p* < 0.0001 vs. control group (Tukey’s test), *n* = 24 from three independent experiments.

The design and discovery of new anticancer agents showing selectively toxicity to tumor cells without affecting normal cells is still a priority goal for cancer therapy. Consistent with this concept, we further analyzed the cytotoxic activity of IMP against normal cells (HSF), larynx cancer, and rhabdomyosarcoma cells using lactate dehydrogenase (LDH) assay, which measures cell membrane integrity. Compared with control cells, no cytotoxic effects (LDH leakage from the cytoplasm into the culture medium) were observed on normal and cancer cells in the concentration range of 1 to 100 µM. This phenomenon has been solely shown at the highest concentration of imperatorin. However, LDH release in normal cells was lower than that of cancer cells (Figure 2).

Taken together, our results indicated that rhabdomyosarcoma and larynx cancer cells showed the best response to IMP treatment, and thus were further used to explore a possible underlying IMP-mediated mechanism of anti-cancer action on the cellular level.

### 2.2. IMP Induced Apoptosis in Human Rhabdomyosarcoma and Larynx Cancer Cells

**Figure 3:**
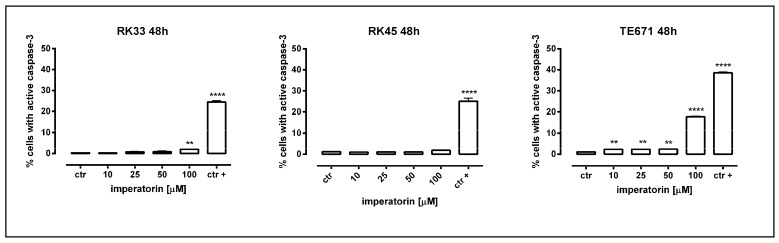
Imperatorin induces apoptotic cell death in human rhabdomyosarcoma and larynx cancer cells. RK33, RK45, and TE671 cells were incubated for 48 h with different concentrations of imperatorin (10–100 µM) and camptothecin (5 µM) as a positive control (ctr+) and analyzed by flow cytometry. The values present the percentage of cells with active caspase-3 are summarized in Table 1. The results are presented as means ± SEM, *n* = 5 from five independent experiments (** *p* < 0.01; **** *p* < 0.0001 versus control, Tukey’s test).

**Figure 4:**
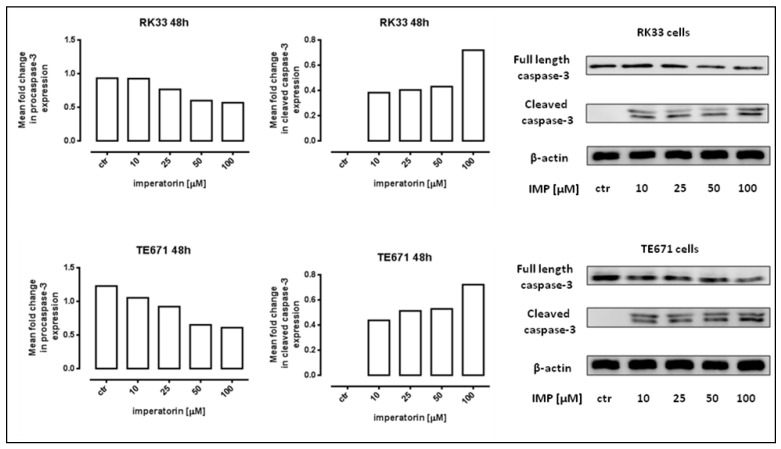
Western blotting analysis of the full length and cleaved caspase-3 activities following 48h incubation of RK33 and TE671 cells with either culture medium alone (control) or imperatorin (IMP) (10–100 µM). β-actin was used as a gel loading control. The right panel shows representative blots; the left panel shows a densitometry analysis of the bands.

Next, we determined whether the antiproliferative of IMP was associated with apoptosis induction. Our study showed that IMP triggered apoptotic cell death in TE671 cells in a dose–dependent manner, present already at 10 µM (2.3% of apoptotic cells, ** *p* < 0.01, and significantly increased (17.8% of apoptotic cells, **** *p* < 0.0001) after the incubation with 100 µM of IMP. The treatment of RK33 and RK45 cells with 10 µM of IMP induced apoptosis to level similar to control cells. At the highest concentration of IMP, we only observed a statistically significant increase in the amount of active caspase-3 positive RK33 cells. As shown in Figure 3, the percentage of apoptotic RK33 cells after treatment with 100 µM IMP was found to be 2.033%; ** *p* < 0.01. These results were further confirmed by detection of cleaved caspase-3 (activated caspase-3) by Western blot analysis. The proteolytic cleavage of caspase-3 protein into two subunits is a general marker for cells undergoing apoptosis [20]. As shown in Figure 4, IMP at concentrations of 10–100 µM induced an increase in the active form of caspase-3 in both RK33 and TE671 cells, which was consistent with flow cytometry data analysis. Furthermore, the activation of caspase-3 was accompanied by a decrease in the procaspase-3 levels in cancer cells. Given that caspase-3 is a key executioner of the apoptotic machinery [21], our results clearly demonstrate that IMP was able to trigger apoptosis through the caspase-dependent mechanism.

### 2.3. IMP Inhibited Cell Cycle Progression in the G1 Phase in Human Rhabdomyosarcoma and Larynx Cancer Cell Lines

**Figure 5:**
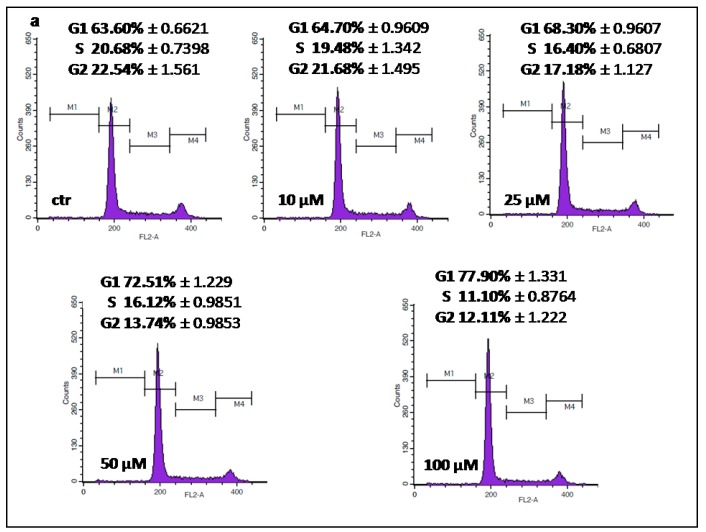

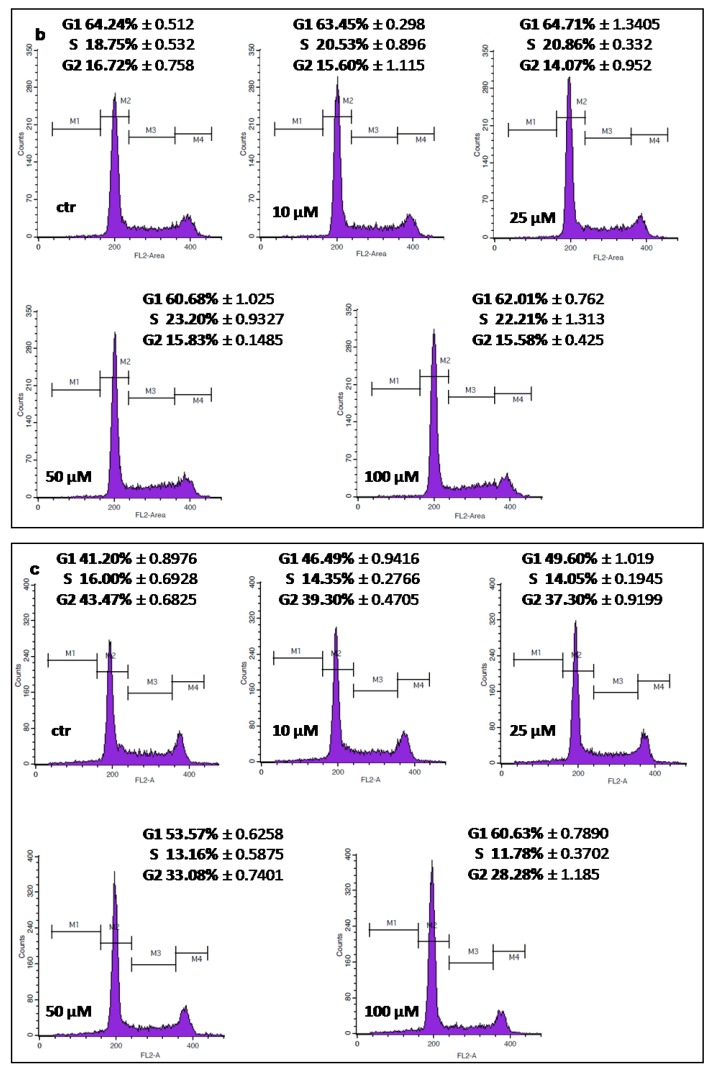
Representative histogram plots of the flow cytometry analysis (DNA content) of RK33 (**a**), RK45 (**b**), and TE671 (**c**) cells upon imperatorin treatment. Results are presented as mean ± SEM, *n* = 9 per concentration from three independent experiments.

To further analyze the mechanism by which IMP inhibited proliferation of rhabdomyosarcoma and larynx cancer cell lines, we performed cell cycle analysis by means of flow cytometry. Fluorescence-activated cell sorting (FACS) analysis revealed that treatment of TE671 and RK33 cells with IMP (10–100 µM) influenced cell cycle progression, inducing G1 phase cell cycle arrest in both cancer cell lines. After incubation with 100 µM IMP, the population of cells in G1 phase increased to 77.90% (RK33) and 60.63% (TE671) in comparison with the control (63.60% and 41.20%, respectively). These changes were accompanied by a reduction of cell numbers in the S/G2 phase (Figure 5a,c). There were no apparent changes in the cell cycle progressionin RK45 cells treated with IMP (Figure 5b).

### 2.4. IMP Altered p21 and Cyclin D1 Expression on Protein and mRNA in Human Rhabdomyosarcoma and Larynx Cancer Cell Lines

**Figure 6:**
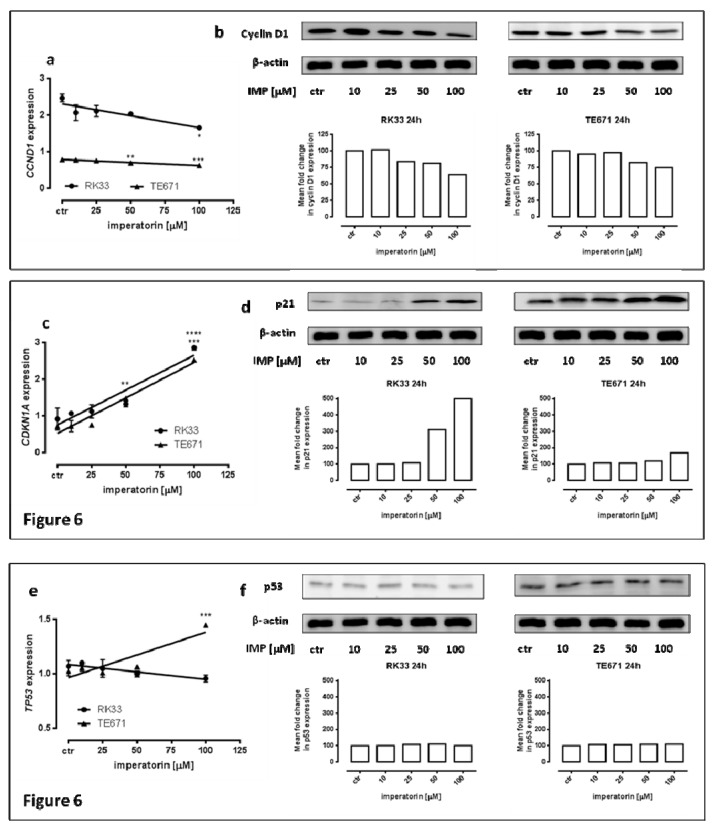
p21, cyclin D1, and p53 expression on protein and mRNA level in human rhabdomyosarcoma and larynx cancer cell lines. Quantification of *CCND1* (**a**) and *CDKN1A* (**c**) and *TP53* (**e**) genes expression by means of qPCR in RK33 and TE671 cells exposed (24 h) to imperatorin (25–100 µM) compared with controls (* *p* < 0.05; ** *p* < 0.01; *** *p* < 0.001; **** *p* < 0.0001; *n* = 9 measurements from three separate experiments, Tukey’s test). Western blots were normalized to β-actin and densitometric analysis of the bands was performed by ImageJ program (**b**,**d**,**f**).

Parallel to the changes in cell cycle progression, we observed a significant effect of IMP on the expression of genes (*TP53*, *CDKN1A*, and *CCND1*) coding for the key cell cycle regulatory proteins p53, p21^Waf1/Cip1^, and cyclin D1, respectively, in TE671 and RK33 cancer cell lines. As shown in Figure 6, the expression of *CDKN1A* was equally upregulated in a dose-dependent manner in both cancer cell lines and increased threefold (RK33 *** *p* < 0.001; TE671 **** *p* < 0.0001) versus the control in the presence of 100µM IMP. There was a slight downregulation of *CCND1* expression in both TE671 and RK33 cell lines. These results were further confirmed by Western blot analysis. Quantitative real-time expression analysis (qPCR) also revealed a statistically significant increase in *TP53* mRNA expression in TE671 cells (*** *p* < 0.001), but not in R33 cells.

## 3. Discussion

Despite significant progress in understanding of cancer biology and intensive anticancer research to improve standard therapy and develop new chemotherapeutic agents, survival rates for patients are still poor. The failure of cancer therapies contributed to a renewed interest in traditional medicine to indentify natural active compounds from plants as a promising chemopreventive or chemotherapeutic candidates [22], following the discovery of taxol [23] and its success in the treatment of many cancer types, including ovarian cancer, breast cancer, colorectal cancer, squamous cell carcinoma of urinary bladder, head and neck cancers, and small-cell and non-small-cell lung cancers (NSCLCs). Coumarins and coumarin-related compounds have been under extensive studies and have proved to show significant anticancer activity against various cell lines in vitro [4] and many tumors in vivo [24]. In our previous studies, we demonstrated that simple coumarin derivatives, including umbelliferone, scoparone, and herniarin, inhibited proliferation and migration of laryngeal cancer cells [25]. Moreover, the other simple coumarin, osthole, exhibited a growth inhibitory effect on human larynx and rhabdomyosarcoma cell lines [26] through induction of apoptosis and cell cycle arrest. In the present study, we focused on structural analogues of furanocoumarins—IMP, IPP, XN, and XNO—in order to assess their anti-cancer activity against human lung, rhabdomyosarcoma, and larynx cancer cell lines. Our experiments revealed that, among the studied linear furanocoumarins, IMP, which possesses the isopentenyloxy group, showed the greatest antiproliferative activity against larynx and rhabdomyosarcoma cells. Furthermore, XNO with an 8-hydroxy group had more potent activity against A549 cells and both studies of larynx cancer cell lines than XN and IPP containing an 8-methoxy group. On the other hand, the functional 5-methoxy group seemed to decrease cytotoxic efficiency of IPP in comparison with XN towards A549, H2170, RK45, and TE671 cells. The present results are consistent with previous reports suggesting that the presence of substituents attached to the benzene ring at positions C-5 and C-8 may be important for enhancing the anticancer properties of furanocoumarins [22,27,28].

**Figure 7:**
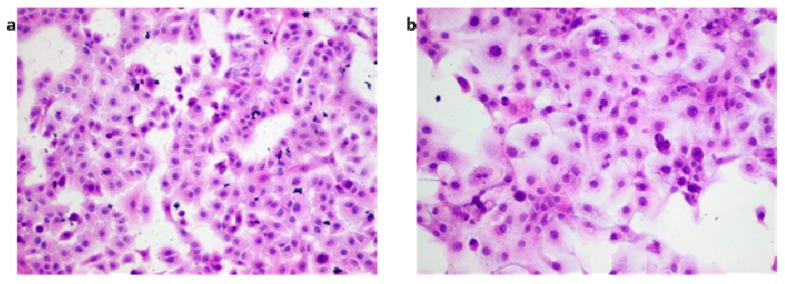
Hematoxylin–eosin (H–E) staining for laryngeal cancer cell lines RK33 (**a**) and RK45 (**b**).

The ability of IMP to inhibit cancer cell growth has been well demonstrated against several malignant cell types, including liver, colon, cervical, lung, and leukemia [29]. However, its specific mechanism of action in rhabdomyosarcoma and larynx cancer is still unclear. There is only one report demonstrating inhibition of laryngeal cancer cell line Hep2 by IMP treatment, probably owing to a decrease in Hsp27 and Hsp72 expressions [30], whereas there are no reports showing IMP influence on rhabdomyosarcoma cells. In the current study, we used laryngeal cancer cell lines derived from tumors of the same histological type arising in different patients [31]. Both analyzed larynx cancer cell lines exhibit similar features, corresponding to squamous cell carcinoma. In general, cells are medium-sized, polygonal, and have round to oval nuclei with a few small nucleoli. An abundant pale or weakly eosinophilic cytoplasm and single giant multinucleated cells were observed in RK45 cell line, whereas RK33 form adenoid-like structures with some more spindle-shaped cells (Figure 7). We found that RK33 and RK45 cells showed unlike biological potential, which was manifested in both a differential cell growth rate and different response to IMP, showing the importance of personalized therapy in this type of cancer. We found that IMP decreased the viability of larynx cancer and rhabdomyosarcoma cells by promoting apoptosis, following or concurrent with cell cycle arrest at the G1/S phase and reduction of cell numbers in the S/G2 phase. The two most affected RK33 and TE671 cell lines display similar sensitivity on 50 µM IMP concentration, resulting in 75.26% and 78.19% inhibition of cells viability, respectively. Apoptosis assay showed the same level of caspase-3 activation (2.03% vs. 2.49%) at a 50 µM IMP concentration in TE671 cells, and 100 µM IMP concentration in RK33 cells (Table 1). Although the apoptotic process assessed by FACS analysis seems to be weekly activated, in Western blot analysis, it was evident, as demonstrated by dose-dependent decrease of pro-caspase 3 form, being mirrored by an increase of cleaved caspase form. The above mentioned IMP concentrations (50 µM in TE671 cells, and 100 µM in TE671 cells) also evoked very similar changes in the inhibition of the cell cycle progression—a decrease in the amount of cells in the G1 phase (14.3% vs. 12.37%) and G2 phase (10.43% vs. 10.39%). This phenomenon was accompanied by suppression of *CCND1* and induction of *CDKN1A* genes expression, coding regulatory proteins of cell cycle progression cyclin D1 and p21^WAF1/CIP1^, respectively. Similar findings have been shown in cervical carcinoma (HeLa), where IMP significantly inhibited the tumor necrosis factor (TNF)-α-induced expression of cyclin D1 [32] and in HT-29 colon cancer cells affected by IMP treatment, resulting in cell cycle arrest in the G1 phase and apoptosis induction [33]. The discrepancies in IMP-mediated apoptosis induction in TE671 (high) and RK33 and RK45 (low) observed in our present study are yet to be solved and require further studies. We found that cell line TE671 is likely a subclone of RD cells, the most commonly used cell line in rhabdomyosarcoma (RMS) research [34]. RD cells carry a p53 missense mutation [35]. Such a P53 mutant is known to impede the cleavage of caspase-3 through directly binding to that protein [36]. The p53 status of RK33 cells in unknown. Therefore, it can be assumed that apoptosis is independent of p53 activity. Moreover, we demonstrated an IMP-mediated increase of p21^Waf1/Cip1^ expression in both TE671 and RK33 cell lines, suggesting that this phenomenon could be responsible for the observed inhibition of the cell cycle progression, especially that other cell cycle-dependent proteins (*CCND1* and *TP53*) yielded different expression patterns in these cell lines. However, as observed in our studies, an increase of p21^Waf1/Cip1^ expression and its potential role for IMP-mediated inhibition of cancer cells proliferation should be later validated by functional in vitro and in vivo studies.

In the search for safe and effective new molecules that could become drugs, the evaluation of pharmacokinetic parameters of various herbal substances has great importance. In the last years, some pharmacokinetic studies regarding pure imperatorin or *Angelica dahurica* extract, containing IMP as the major active component, have been reported. Published pharmacokinetic data vary widely and showed that the maximum plasma concentration (C_max_) of IMP ranged from 0.04 µg/mL to 1.06 µg/mL (0.15 µM to 3.9 µM) following the oral administration of pure IMP at doses of 6.25–80 mg/kg [37,38]. In our in vitro studies, it was shown that IMP inhibited viability of cancer cells at a relatively high concentrations, which cannot be achieved in vivo. However, what is interesting, is that moderate concentrations of IMP used in the present study (10–50 µM) were enough to observe some mechanisms by which IMP exerted its anticancer activity, including apoptosis induction and cell cycle arrest. Moreover, the above concentrations of IMP were close to mean C_max_ values of IMP, such as 2587 ng/mL (8.2–10.8 µM) and 12.921 ng/mL (47 µM), after administration of a single dose of 4.5 g/kg (equivalent to 74.1 mg/kg IMP) and 6 g/kg *A. dahurica* extract, respectively [39,40]. It is worth mentioning that the root of *A. dahurica* is frequently used as a food additive and its average daily consumption in China is around 30 g (equivalent to ~120–300 mg IMP) [41]. *A. dahurica* is also used in traditional Chinese medicine for the treatment of headache, toothaches, asthma, coryza, hypertension, vitiligo, psoriasis, acne, herpes zoster virus, and freckles [42].

Taking into consideration that there is not an effective specific chemotherapy treatment dedicated to laryngeal squamous cell carcinoma (except widely used docetaxel, cisplatin, and 5-fluorouracil [43]), and as IMP seems to be non-toxic for normal cells (HSF), our results could open a new direction in the search for effective anti-cancer agents.

## 4. Materials and Methods

### 4.1. Plant Material

The fruits of *Angelica archangelica* L. (Apiaceae) were collected in August 2011, whereas fruits of *Pastinaca sativa* L. (Apiaceae) were collected in August 2010, both in Medicinal Plant Garden, Medical University in Lublin, Poland. Plants were botanically identified by specialist Dr Stanisław Kwiatkowski and a voucher specimen (no. B2/7e9 and 17/20, respectively) is stored in the Department of Pharmacognosy with Medicinal Plant Unit, Medical University of Lublin.

### 4.2. Extraction and Isolation

The air-dried and powdered fruits of *A. archangelica* were extracted exhaustively under reflux with methanol (100 mL of solvent each time, 30 min), whereas the same procedure, but with dichloromethane as a solvent, was applied for air-dried fruits of *P. sativa*. The extracts were evaporated to dryness, and then separated using HPCCC (Spectrum, Dynamic Extractions, Slough, UK) equipped with both analytical and semipreparative coil with 22 and 137 mL capacity, respectively, according to a previously published method [44].

Briefly, different ratios of heptane/ethyl acetate/methanol/waterwere tested. The partition coefficients K_D_ of target compounds in the crude extract were determined by HPLC. Four milliliters of two-phase solvent systems tested were prepared and shaken vigorously with a small portion of crude extracts. After fully separating, 1 mL of each layer was removed, evaporated, dissolved in 1 mL of methanol, and then analyzed by HPLC. The K_D_ values of the target compounds were calculated according to the equation K = AU/AL, where AU and AL were the peak areas of target compound in the upper stationary and lower mobile phases, respectively. The partition coefficient should lie within the approximate range of 0.5 < K < 2.0. A smaller K_D_ value results in a loss of peak resolution, while a larger value produces excessive band broadening [18]. Finally, a two-phase solvent system composed of heptane, ethyl acetate, methanol, and water (1:1:1:1) was used for separation in reversed phase mode. After injection of 800 mg of crude methanolic extract from *A. angelica*, 120 mg of pure IMP was isolated in less than 25 min (K_D_ = 1.85; purity 98%). The same solvent system was used to purify XN from fruits of *P. sativa*. Injection of 800 mg of crude oily extract resulted in obtaining 30 mg of XN (K_D_ = 0.92, purity 98%) [9]. In both cases, the upper stationary phase was pumped into the semipreparative column at a flow rate of 6.0 mL/min in head to tail mode. After filling the coil, the apparatus was rotated at 1600 rpm, and then the mobile lower phase was pumped. The separation temperature was set at 25 °C. The effluents were continuously monitored at 254 nm. After hydrodynamic equilibrium was reached, the crude extract solution was injected using the manual 6 mL sample loop. One-minute fractions were collected and monitored by HPLC. Fractions containing a single peak were pooled together and evaporated. The identity and purity of isolated compounds were confirmed by HPLC-DAD and NMR analyses. A 600 MHz Bruker spectrometer was used for 1D-NMR (^1^H-, and ^13^C-NMR) spectra.

**Figure 8:**
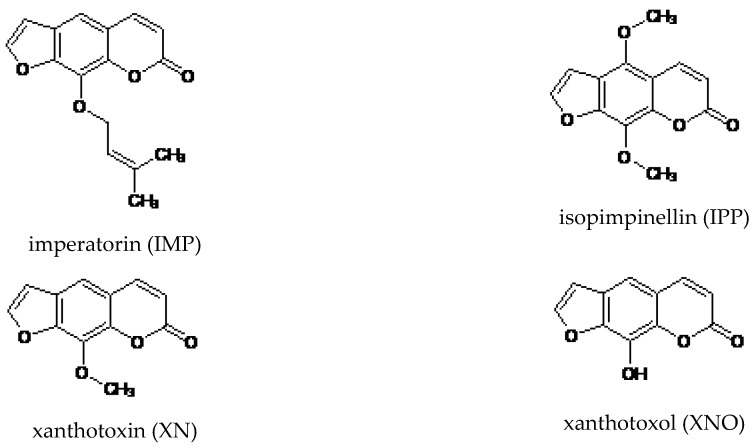
The chemical structures of analyzed coumarins.

IPP and XNO, as structure analogues of furanocoumarins, were delivered from Sigma Aldrich. Stock solution of studied furanocoumarins was prepared in DMSO (Sigma Chemicals, St. Louis, MO, USA). The chemical structures of analyzed coumarins are presented in Figure 8.

### 4.3. Cell Lines

Human rhabdomyosarcoma (TE671) and lung cancer cell lines (A549, H2170 and H1299) were obtained from the American Type Culture Collection (ATCC). Human larynx cancer cells (RK33 and RK45) were derived from patients with diagnosed larynx squamous cell carcinomas. Cancer tissue was removed from the larynx after total laryngectomy and established as a stable cell line, as previously described [31]. Normal human primary fibroblast culture (HSF) was obtained by the outgrowth technique from skin explants of a young person, using a method routinely ongoing in our lab (Local Ethical Committee permission No KE0254/298/2015). TE671 cell line was maintained in nutrient mixture F-12 Ham culture medium (Sigma Chemicals, St. Louis, MO, USA). A549 was grown in a 1:1 mixture of DMEM and nutrient mixture F-12 Ham (Sigma Chemicals, St. Louis, MO, USA). RPMI 1640 medium (Sigma Chemicals, St. Louis, MO, USA) was used for culture of H2170, H1299, RK33, and RK45 cell lines and HSF. All culture media were supplemented with 10% fetal bovine serum (FBS) (Sigma Chemicals, St. Louis, MO, USA), penicillin (100 µg/mL) (Sigma Chemicals, St. Louis, MO, USA), and streptomycin (100 µg/mL) (Sigma Chemicals, St. Louis, MO, USA). Cultures were kept at 37 °C in a humidified atmosphere of 95% air and 5% CO_2_.

### 4.4. Cell Viability Assessment

Cancer cells were placed on 96-well plates (Nunc, Roskilde, Denmark) at a density of 1 × 10^4^ cells/mL. The next day, the culture medium was removed and cells were exposed to serial dilutions of linear furanocoumarins in fresh culture medium. Cell viability/proliferation was assessed after 72 h by means of the MTT method, in which the yellow tetrazolium salt (MTT) is metabolized by viable cells to purple formazan crystals. Cells were incubated for 3 h with MTT solution (5 mg/mL, Sigma). Formazan crystals were solubilized overnight in sodium dodecyl sulfate (SDS) buffer (10% SDS in 0.01 N HCl), and the product was determined spectrophotometrically by measuring absorbance at 570 nm wavelength using Infinite M200 Pro microplate reader (Tecan, Männedorf, Switzerland).

### 4.5. Cytotoxicity Assessment—LDH Assay

Optimized amounts of HSF, RK33, RK45, and TE671 (1 × 10^5^ cells/mL) were placed on 96-well plates (Nunc). The next day, cells were washed in phosphate-buffered saline (PBS) and then exposed to increasing concentrations of imperatorin in fresh culture medium containing 1% FBS. The cytotoxicity was estimated based on the measurement of cytoplasmic lactate dehydrogenase (LDH) activity released from damaged cells after 24 h of exposure to imperatorin. LDH assay was performed according to manufacturer’s instruction (Cytotoxicity Detection KitPLUS (LDH) (Roche)). Briefly, 50 µL of cell medium was collected from each well, after which 50 µL of the reaction mixture (freshly prepared) was added and incubated for 30 min at room temperature (RT). Finally, 25 µL of stop solution was added to each well on the 96-well plate. Absorbance was measured at two different wavelengths, one being the “measurement wavelength” (492 nm) and the other “reference wavelength” (690 nm) using Infinite M200 Pro microplate reader (Tecan). Maximum LDH release (positive control) was achieved by addition of lysis buffer to untreated control cells. The average values of the culture medium background were subtracted from all values of experimental wells and the percentage of death cells was calculated in relation to the maximum LDH release.

### 4.6. Assessment of Apoptosis

Human rhabdomyosarcoma and larynx cancer cells were placed on six-well plates (Nunc) at a density of 1 × 10^5^/mL. The next day, the medium was replaced with fresh medium containing different concentrations of imperatorin (10, 25, 50, and 100 µM) for 48 h. After that, cells were harvested and washed twice with PBS. Next, cells were fixed and permeabilized using the cytofix/cytoperm solution according to the manufacturer’s instructions of Phycoerythrin (PE) Active Caspase-3 Apoptosis Kit (BD Pharmingen). Finally, cells were washed twice in the perm/wash buffer prior to intracellular staining with PE-conjugated anti-active caspase-3 monoclonal rabbit antibodies. Labelled cells were analyzed by flow cytometer FACSCalibur (Becton Dickinson, San Jose, CA, USA), operating with CellQuest software to quantitatively assess the caspase-3 activity.

### 4.7. Cell Cycle Analysis

Experiments were performed using the FACSCalibur^TM^ flow cytometer (BD Biosciences), equipped with a 488nm argon-ion laser. For cell cycle analysis, cells were treated with different concentrations of imperatorin for 24 h and then fixed/permeabilized in ice-cold 80% ethanol at −20 °C for 24 h. After fixation, the cells were stained with propidium iodide utilizing the PI/RNase Staining Buffer (BD Biosciences) according to the manufacturer’s instructions. The acquisition rate was at least 60 events per second in low acquisition mode and at least 10.000 events were measured. Cell cycle analysis was performed by using a noncommercial flow cytometry analyzing software, Cylchred Version 1.0.2 for Windows (source: University of Wales), and WinMDI 2.9 for Windows (source: facs.scripps.edu/software.html). The cells were acquired and gated by using dot plot FL-2 Width (X) versus FL-2 Area (Y)-gate to exclude aggregates, and analyzed in histograms displaying fluorescence two-area (yellow-orange fluorescence: 585 nm).

### 4.8. Protein Extraction and Western Blot Analysis

Tumour cells were placed on a six-well plates (Nunc) at a density of 1 × 10^5^ cells/mL (RK33) and 5 × 10^4^ cells/mL (TE671). On the following day, the culture medium was removed and the cells were exposed to selected concentrations of IMP. After treatment, the cells were harvested, washed twice in ice-cold PBS, lysed in Radioimmunoprecipitation Assay (RIPA) buffer (Sigma) supplemented with protease inhibitor cocktail (Sigma), and centrifuged at 14,000× *g* for 10 min at 4 °C. The total protein concentrations were quantified spectrophotometrically using the Protein Assay Kit (Bio-Rad Laboratories, Hercules, California, USA) according to the manufacturer’s instructions. For Western blot analysis, the protein extracts were solubilized in Laemmli buffer (0.19 M Tris-HCl, pH 6.8, containing 30% glycerol, 3% SDS, 0.015% bromophenol blue, and 3% β-mercaptoethanol) and boiled for 5 min at 100 °C. Then, equal amounts of the protein lysates were electrophoresed on SDS-PAGE and electroblotted to polyvinyl difluoride (PVDF) membranes (Merck Chemicals, Darmstadt, Germany). Next, the membranes were blocked in Tris Buffered Saline (TBS) with 5% non-fat dry milk and 0.05% Tween 20, pH 7.5, for 2 h at room temperature (RT), and incubated overnight at 4 °C with the following primary antibodies: caspase-3, cleaved caspase-3 (Asp175), cyclin D1, p21, and p53 (Cell Signaling Technology, Beverly, MA, USA). Antibody dilutions were prepared according to the product data sheet. On the following day, the membranes were washed and then incubated with a horseradish peroxidase-labeled secondary antibody (Cell Signaling Technology) for 1 h at RT. Finally, the membrane was visualized using a Lumi-Light Western Blotting Substrate (Roche, Mannheim, Germany) according to the manufacturer’s instructions. Subsequently, stripping buffer (62.5 mM Tris-HCl, pH 6.8, with 100 mM β-mercaptoethanol and 2% SDS) was used to remove bound antibodies and the membranes were reprobed with anti-β-actin (Cell Signaling) used as a load control. Densitometric measurement of protein level was performed using the GeneTools (Syngene). Obtained data were normalized to β-actin expression.

### 4.9. RNA Isolation and cDNA Synthesis

RK33 and TE671 cells were incubated on six-well plates with the selected concentrations of imperatorin for 24 h. Total RNA from the cells was isolated and digested with DNase using High Pure RNA Isolation Kit (Roche, Mannheim, Germany) following the manufacturer’s instructions. Briefly, the cells were resuspended in PBS and lysed in lysis/binding buffer. The cell lysates were transferred to a high pure filter tube and centrifuged at 8000× *g* for 15 s. Next, the RNA was incubated with DNase for 15 min at RT. The resulting RNA was rinsed once in wash buffer I, rinsed twice in wash buffer II, centrifuged at 8000× *g* for 15 s, and finally eluted using elution buffer. The RNA concentration was determined spectrophotometrically with UV/visGenesys 10S spectrophotometer at 260/280 nm (Thermo Fisher Scientific). Then, 3 μg of total RNA was reverse transcribed for 30 min at 50 °C using oligo(dT) primer and Transcriptor High Fidelity cDNA Synthesis Kit (Roche), followed by 5 min enzyme inactivation at 85 °C according to the manufacturer’s instructions.

### 4.10. qPCR

qPCR analyses were performed using LightCycler^®^ 480 II instrument (Roche). Analyses were conducted utilizing Universal ProbeLibrary (UPL) hydrolysis probes specific for *TP53*, *CCND1*, and *CDKN1A* genes labeled with carboxyfluorescein (FAM) in duplex with the probe for reference gene *GAPD* labeled with Yellow 555 (Roche). The primers and probe sets were as follows: *TP53* (For 5′-CCCCAGCCAAAGAAGAAAC-3′, Rev 5′-AACATCTCGAAGCGCTCAC-3′, Probe 5′-GGATGGAG-3′); *CCND1* (For 5′-GAAGATCGTCGCCACCTG-3′, Rev 5′-GACCTCCTCCTCGCACTTCT-3′, Probe 5′-TGCTGGAG-3′); *CDKN1A* (For 5′-TCACTGTCTTGTACCCTTGTGC-3′, Rev 5′-GGCGTTTGGAGTGGTAGAAA-3′, Probe 5′-CCTGGAGA-3′); *GAPD* (For 5′-CTCTGCTCCTCCTGTTCGAC-3′, Rev 5′-GCCCAATACGACCAAATCC-3′, Probe 5′-CTTTTGCGTCGC-3′). Amplification was performed in 10 μL of reaction mixture containing cDNA amount corresponding to 12.5 ng of total RNA, 1x LightCycler^®^ 480 Probes Master (Roche), and an appropriate set of 0.4 μM primers and 0.2 μM UPL hydrolysis probes for each target and reference duplex. After 10 min of initial denaturation at 95 °C, cDNA was amplified in 45–55 cycles consisting of 10 s denaturation at 95 °C, 30 s annealing at 60 °C, and 10 s elongation at 72 °C. Obtained fluorescence data were calculated using the relative quantification method with efficiency correction.

### 4.11. Statistics

GraphPad Prism 6 Statistic Software was used for statistical analysis. The calculations were done by one-way analysis of variance (ANOVA) test for multiple comparisons followed by Tukey’s significance test. Data are expressed as the mean standard error (SEM) (* *p* < 0.05, ** *p* < 0.01, *** *p* < 0.001, **** *p* < 0.0001). IC_50_ was calculated using computerized linear regression analysis of quantal log dose-probit functions according to the method of Litchfield and Wilcoxon [45].

## Figures and Tables

**Table 1:** The percentage of cancer cells positive for active caspase-3 when treated with IMP for 48 h.

	ctr	ctr+	10 µM	25 µM	50 µM	100 µM
**RK33**	0.3100	24.51	0.2600	0.7533	0.8400	2.033
**RK45**	1.190	25.12	1.000	1.090	1.157	1.870
**TE671**	1.067	38.61	2.345	2.348	2.497	17.83
